# Correlates of uptake of optimal doses of sulfadoxine-pyrimethamine for prevention of malaria during pregnancy in East-Central Uganda

**DOI:** 10.1186/s12936-020-03230-8

**Published:** 2020-04-15

**Authors:** Mbonye K. Martin, Kirwana B. Venantius, Ndugga Patricia, Kikaire Bernard, Baleeta Keith, Kabagenyi Allen, Asiimwe Godfrey, Twesigye Rogers, Kadengye T. Damazo, Byonanebye M. Dathan

**Affiliations:** 1 Department of Population Studies, School of Statistics and Planning, College of Business and Management Sciences, Makerere University, Kampala, Uganda; 2 Mildmay, Kampala, Uganda; 3 Department of Pediatrics and Child Health, School of Medicine, College of Health Sciences, Makerere University, Kampala, Uganda; 4 Uganda Virus Research Institute, Entebbe, Uganda; 5 University Research Co., LLC, Jinja, Uganda; 6 Reformed Theological College, Kampala, Uganda; 7 African Population and Health Research Centre, Nairobi, Kenya; 8 Department of Community and Behavioral Sciences, School of Public Health, College of Health Sciences, Makerere University, Kampala, Uganda

**Keywords:** Intermittent preventive treatment during pregnancy, Malaria, Uganda, Demographic and health survey

## Abstract

**Background:**

In 2012, the World Health Organization recommended that pregnant women in malaria-endemic countries complete at least three (optimal) doses of intermittent preventive treatment (IPTp) using sulfadoxine-pyrimethamine (SP) to prevent malaria and related adverse events during pregnancy. Uganda adopted this recommendation, but uptake remains low in East-Central and information to explain this low uptake remains scanty. This analysis determined correlates of uptake of optimal doses of IPTp-SP in East-Central Uganda.

**Methods:**

This was a secondary analysis of the 2016 Uganda Demographic Health Survey data on 579 women (15–49 years) who attended at least one antenatal care (ANC) visit and had a live birth within 2 years preceding the survey. Uptake of IPTp-SP was defined as optimal if a woman received at least three doses; partial if they received 1–2 doses or none if they received no dose. Multivariate analysis using multinomial logistic regression was used to determine correlates of IPTp-SP uptake.

**Results:**

Overall, 22.3% of women received optimal doses of IPTp-SP, 48.2% partial and 29.5% none. Attending ANC at a lower-level health centre relative to a hospital was associated with reduced likelihood of receiving optimal doses of IPTp-SP. Belonging to other religious faiths relative to Catholic, belonging to a household in the middle relative to poorest wealth index, and age 30 and above years relative to 25–29 years were associated with higher likelihood of receiving optimal doses of IPTp-SP.

**Conclusions:**

In East-Central Uganda, uptake of optimal doses of IPTp-SP is very low. Improving institutional delivery and household wealth, involving religious leaders in programmes to improve uptake of IPTp-SP, and strengthening IPTp-SP activities at lower level health centers may improve uptake of IPTp-SP in the East-Central Uganda.

## Background

Malaria infection in pregnancy is a major public health problem and is associated with adverse pregnancy and birth outcomes. Malaria during pregnancy is not only deleterious to the woman as it can lead to maternal anaemia, miscarriage and stillbirth, but it also puts her fetus at increased risk of adverse outcomes, such as preterm delivery, low birth weight, and intrauterine growth restriction [1].

In 2015, malaria in pregnancy was estimated to be responsible for approximately 400,000 cases of maternal anaemia and 15% of maternal deaths globally [2]. The burden and impact of malaria is highest in sub-Saharan Africa (SSA), where fertility rates and malaria endemicity are high and health systems are weak [3]. In SSA alone, malaria in pregnancy is responsible for 10% of maternal deaths [3], 20% of stillbirths and 11% of all new-born deaths [4].

To reduce the burden and impact of malaria, the World Health Organization (WHO) recommends a package of interventions for prevention and control of malaria especially during pregnancy. These include use of insecticide-treated nets (ITNs), indoor residual spraying (IRS) with a mosquito insecticide, appropriate malaria case detection and treatment and taking appropriate dose of intermittent preventive treatment (IPTp) with sulfadoxine-pyrimethamine (SP), also known as Fansidar^®^, for malaria prevention during pregnancy [5, 6]. IPTp-SP is an anti-malarial medicine given to all pregnant women during routine antenatal care (ANC) visits to protect them from the risk of getting malaria and related adverse outcomes and treatment of placental malaria.

Since 2012, the WHO recommends that IPTp-SP is given to all pregnant women in malaria-endemic countries who attend ANC, intermittently one month apart starting at the earliest opportunity in the second trimester with at least three (*optimal*) doses administered until the end of the pregnancy period [7–9]. This recommendation was made following evidence showing that receiving a minimum of three doses of IPTp-SP during pregnancy was associated with superior benefits, such as fewer low birth weight infants and higher mean weight at birth compared to two doses [10, 11] previously recommended by the WHO and one or no IPT-SP doses [10, 12, 13]. Even amidst recent concerns on increasing resistance, IPTp-SP has been shown to be associated with reduced maternal anaemia and perinatal mortality rates in SSA, including areas with more than 90% resistance [1, 7, 14]. This WHO recommendation has been endorsed and adopted by at least 37 countries with a high malaria burden, including Uganda [15].

Despite the visibly considerable investments in malaria prevention and overwhelming evidence of efficacy and impact of IPTp-SP, implementation remains a challenge and uptake of IPTp-SP remains generally low in SSA [16], especially in countries with high malaria endemicity [17]. A synthesis of data from national surveys done in 2012 indicate that, even prior to adoption of the current IPTp-SP policy, coverage of IPTp-SP was a still challenge in SSA. Results from this synthesis show that 39 of the 45 countries studied had a malaria policy, and that in these 39 countries only 25% of pregnant women received optimal doses of IPTp-SP, with coverage reported lowest in areas of high-intensity malaria transmission. In spite of reported high (77%) ANC attendance, IPTp-SP coverage rates were below 10% in 36% of the countries, below 25% in 61% of the countries and below 50% in 77% of the countries. Only three countries, that is Zambia, the Gambia and Malawi reported IPTp-SP coverage above 80% [18]. In 2017, only 22% of pregnant women received optimal doses of IPTp-SP in 33 SSA countries that reported data on this indicator [5]. During the same time, only 54% received at least one dose and 42% two doses [5]. In Uganda, coverage of optimal doses of IPTp-SP was 17.6% in 2006 [19] and increased to only 26.7% in 2011 [20]. In 2014/2015, it slightly increased to 28% [21] but then reduced to 17% in 2016 [22]. This is comparable to majority of the SSA countries whose coverage of optimal doses of IPTp-SP remains dismal according to a synthesis of results from the most recent country malaria indicator, demographic health and multiple indicator cluster surveys [23]. The results from this synthesis also showed that only Ghana, Zambia, Malawi, Sierra Leonne and Guinea had coverage of optimal doses of IPTp-SP of at least 30% [23].

In 2016, East-Central Uganda, also referred to as Busoga was among the three of the 15 sub-regions with the lowest IPTp-SP coverage in Uganda. East-Central Uganda is an area of high malaria endemicity [24], very low contraceptive use and high fertility and teenage pregnancy rates [22] and very high rate of poverty [25]. In addition to socio-economic, demographic and health seeking factors, low uptake of IPTp-SP is likely as a result of complacence of health workers to prescribe and encourage uptake of IPTp-SP, high costs of accessing malaria prevention services, lack of awareness and late presentation for ANC, weak leadership and frequent stock-outs of medicines [26, 27]. Generally, there is little information to explain the low uptake of IPTp-SP, especially for East-Central Uganda.

To improve IPTp-SP coverage in East-Central Uganda and similar areas, there is need to understand the socioeconomic, demographic and health-seeking behaviour disparities among pregnant women. This study determined factors associated with uptake of optimal doses of IPTp-SP in East-Central Uganda. In addition, this study also sought to understand factors associated with uptake of a partial (1–2) dose of IPTp-SP.

## Methods

### Study design and data source

This was a quantitative observational study that involved secondary analysis of data collected during the 2016 Uganda Demographic Health Survey (UDHS). The 2016 UDHS was a nationally representative cross-sectional sample survey in which 19,588 households participated in the survey. These households were distributed in 696 enumeration areas selected based on the 2014 Uganda national household census. During this survey, a total of 18,506 eligible women aged 15–49 years were interviewed, and the response rate was 97%. The survey was conducted by the Uganda Bureau of Statistics (UBOS) with technical assistance from ICF International. The methodology for the 2016 UDHS has been previously described [22].

### Setting

This study focused on East-Central Uganda (called Busoga in the 2016 UDHS), one of the 15 sub-regions considered for the 2016 UDHS. East-Central Uganda included 10 of the 112 districts in Uganda at the time of the survey. The districts in East -Central Uganda were: Bugiri, Namutumba, Buyende, Iganga, Jinja, Kaliro, Kamuli, Luuka, Mayuge and Namayingo. East-Central Uganda is an area of high year-round malaria transmission that usually peaks during the rainy seasons (March to May and September to December). The 2018 projected population of East-Central is 3.8 million people, of whom 51% are women and 22.7% are women age 15–49 years [28]. ANC attendance and IPTp-SP prescriptions are freely available at the one regional referral hospitals, 12 general hospitals, 18 Health Centre IVs and 112 Health Centre IIIs within the region [29].

### Study sample

**Fig. 1:**
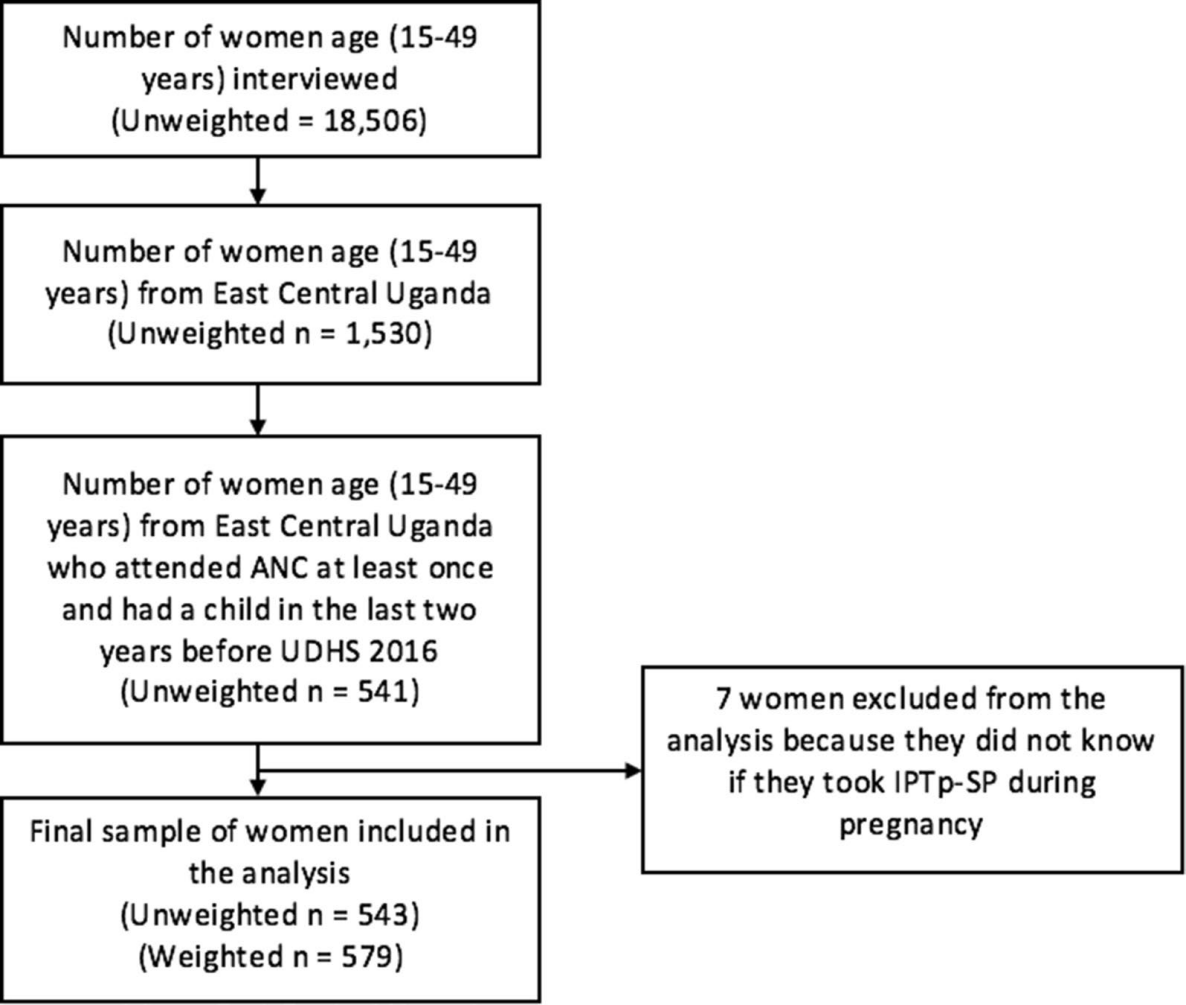
Sample selection criteria

Figure 1 shows the selection criteria for the sample selected for this study. The study sample considered in this analysis comprised of women of reproductive age (15–49 years) from East-Central Uganda who had had a live birth in 2 years preceding the 2016 UDHS and attended ANC at least once. Women who attended ANC were considered since all women attending ANC are supposed to receive IPTp-SP as par the Uganda Clinical guidelines. The final sample comprised 579 women. Detailed description of participant recruitment, eligibility, and study procedures are described elsewhere [22]. Although the survey considered women who had had a birth in 5 years preceding the survey, only women who had had a live birth in 2 years preceding the survey were included to minimize potential recall bias. For these women, the study also considered only ones who had attended ANC at a health facility to increase certainty of information on IPTp-SP uptake.

### Variables

#### Outcome measure

The main outcome of this study was uptake of IPTp-SP. The outcome variable was categorized into three groups: (1) “*None*” if the woman took no dose of IPTp-SP during pregnancy; (2) “*partial*” if the woman took 1–2 doses; and (3) “*optimal*” if the woman took at least three doses.

#### Explanatory variables

After the review of the existing relevant literature, 22 potential socio-economic, demographic, and healthcare utilization covariates associated with IPTp-SP uptake during pregnancy [30–37] were retrieved from the 2016 UDHS dataset for analysis. All the variables were categorical. The 22 covariates and their categories are presented in Table 1.

**Table 1:** List of explanatory variables

No	Variable name	Categories and definition
Demographic		
1	District	Bugiri, Iganga, Jinja, Kamuli, Mayuge, Kaliro, Namutumba, Buyende, Luuka and Namayingo
2	Residence	Urban or rural
3	Marital status	Never married, currently in union, formerly in union
4	Religion	Catholic, Anglican, Muslim, Others. Others: combined 7th day Adventists, pentecostals, born-agains and a few whose religious denominations could not be classified
5	Sex of the household head	Male or female
6	Mother’s age	15-19, 20–24, 25–29, 30–34, 35 and above years
7	Mothers education	No education, primary education, secondary or higher education
8	Parity	1 or 2 pregnancies, 3 or 4 pregnancies, 5 or more pregnancies. Parity was defined as the total number of children ever born
9	Partner’s age	Under 25, 25–35, 35 and above years. A partner was the father of the child or husband to the woman.
10	Partner’s education level	No education, primary education, secondary or higher education
Socio economic		
11	Wealth index	Poorest, poor, middle, richer, richest
12	Ownership of television (TV) set or radio	Yes or no
13	Employment status	Unemployed, employed in agriculture, employed in a non-agriculture setting
Malaria prevention and healthcare utilization		
14	Possession of a bed net	Yes or no
15	Bed net utilization	Yes or no
16	Fever during pregnancy	Yes or no
17	Dwelling sprayed with a mosquito insecticide in the last 6 months	Yes or no
18	Decision maker on seeking health	Woman alone, woman and partner, woman not involved
19	ANC provider	Hospital, lower level health center
20	Timing of 1st ANC visit	Early (first trimester) or late (second and third trimesters)
21	Number of ANC visits	Less than 4 visits or at least 4 visits
22	Perceived distance to the health facility	Problem or not a problem

### Statistical analysis

All the 23 covariates analysed for this study were available in the “woman dataset (IR)” of the 2016 UDHS. All analyses were weighted in order to account for the complex survey design, using the *svy* command in STATA. As thus, only weighted survey results are presented in this paper. This was a 3-level analysis of the 2016 UDHS data. At the first level, descriptive statistics were used to summarize participants’ demographic, socio-economic, and malaria prevention and healthcare services utilization characteristics and results were presented as frequencies and percentages. At the second level, univariate multinomial logistic regression was conducted between the outcome variable and each covariate independently, and covariates that showed statistically significant association at p < 0.05 were included in the multivariate multinomial logistic regression model.

At the third level, multivariate analysis using multinomial logistic regression model was conducted to identify factors associated with IPTp-SP uptake. The multivariate multinomial regression model used generalized estimating equations. Hosmer–Lemeshow test for overall fit of a multinomial logistic regression model was used to test the goodness of fit of the multinomial logistic regression [38]. All statistical analyses were performed using STATA, version 14 (Stata Corporation, College Station, Texas).

For covariates with missing data multiple imputation analysis was done to address missing values. Multivariate imputation with chained equations (MICE) using Ordinal logistic regression was conducted to impute missing values with five iterations, and estimates derived from each iteration were combined using Rubin’s methods [39]. Only estimates of adjusted ratio of relative risk (RRR), their 95% confidence intervals (CI) and p-values derived through multiple imputation analysis are presented.

### Ethics approval and consent to participate

Permission to use the UDHS datasets was sought from the DHS Program website https://www.dhsprogram.com/data/available-datasets.cfm. The ICF IRB reviewed and approved the 2016 Uganda Demographic and Health Survey. The ORC MACRO, ICF Macro, and ICF IRBs complied with the United States Department of Health and Human Services regulations for the protection of human research subjects (45 CFR 46). All participants provided informed verbal consent to participate in the study and for minors, their parents or guardians consented on their behalf. Further details regarding the conduct of the study may be found in the 2016 UDHS report [22].

## Results

### Background characteristics

The background characteristics of the respondents are presented in Table 2. Overall, the mean age was 27.6 (standard deviation of the women: ± 6.8 years, range: 16 to 47 years). Majority of the women lived in rural areas (94%), were of Muslim faith (34.5%), had primary education (63.5%), were currently in union (88%) and were employed in agriculture (42.1%). Additionally, a majority of the women came from households within the poorer (26.4%) wealth index and had parity of at least five children ever born (43.8%) at the time of the survey.

**Table 2:** Background characteristics of the respondents

Characteristic	Number of respondents (n = 579)	Percent (%)
Age of the mother		
16–24 years	205	35.4
25–29 years	142	24.5
30–47 years	232	40.1
Residence		
Urban	47	8.1
Rural	532	91.8
Level of education		
No education	47	8.2
Primary	347	59.9
Secondary or higher	185	31.9
Occupation		
Not employed	161	27.9
Employed–agriculture	249	43.0
Employed–non-agricultural	169	29.1
Religion		
Anglican	205	35.5
Catholic	94	16.2
Muslim	191	33.0
Others	89	15.4
Marital status		
Never been in union	24	4.1
Currently in union	516	89.0
Formerly in union	40	6.9
Wealth index		
Poorest	83	14.4
Poorer	148	25.5
Middle	140	24.1
Richer	138	23.9
Richest	70	12.1
Parity		
1 or 2	192	33.2
3 or 4	135	23.3
5 + s	252	43.5
District		
Iganga	74	12.7
Bugiri	27	4.7
Jinja	76	13.2
Kamuli	82	14.2
Mayuge	82	14.1
Kaliro	27	4.7
Namutumba	60	10.4
Buyende	97	16.7
Luuka	22	3.9
Namayingo	31	5.3

### IPTp-SP coverage

Table 3 presents the overall and district specific percentage distribution of uptake of IPTp-SP during pregnancy in East Central Uganda. Overall, 22.3% of women in East-Central Uganda received optimal doses of IPTp-SP, 48.2% received partial dose and 29.5% no dose at all during pregnancy. Uptake of optimal doses of IPTp-SP during pregnancy was lowest in Kaliro district (8.6%) and highest in Kamuli district (35.4%). More than half (54.7%) of women in Kaliro district that received no dose of IPTp-SP at all, with this district performing the worst on this statistic in the entire East-Central region; Kamuli district (12.0%) had the lowest proportion of women that did not take any dose of IPTp-SP during pregnancy.

**Table 3:** Percentage distribution of uptake of IPTp-SP by respondents’ characteristics

Variable		Number of respondents (n = 579)	IPTp-SP doses			Crude RRR (cRRR)	
			None %	1–2 doses %	3 and more %	1–2 IPTp-SP doses cRRR [95%CI], p-value	3 and more IPTp-SP doses cRRR [95%CI], p-value
		n = 579	% = 29.5	% = 48.2	% = 22.3		
District							
Iganga		74	22.3	55.3	22.4	Ref	Ref
Bugiri		27	39.7	37.8	22.5	0.72 [0.24─2.13], 0.549	0.89 [0.39─2.01], 0.766
Jinja		76	27.1	48.7	24.2	0.38 [0.10─1.35], 0.133	0.57 [0.23─1.38], 0.204
Kamuli		82	12.0	52.6	35.4	1.77 [0.44─7.15], 0.416	2.94 [0.92─9.37], 0.068
Mayuge		82	35.5	41.7	22.8	0.47 [0.16─1.35], 0.159	0.64 [0.37─1.11], 0.111
Kaliro		27	54.5	36.9	8.6	0.27 [0.10─0.75], 0.013	0.16 [0.06─0.39], 0.000
Namutumba		60	27.4	57.1	15.5	0.84 [0.27─2.61], 0.757	0.56 [0.37─0.84], 0.006
Buyende		97	40.9	40.1	19.0	0.40 [0.15─1.06], 0.067	0.46 [0.20─1.05], 0.066
Luuka		22	22.2	53.5	24.3	0.97 [0.33─2.83], 0.954	1.08 [0.40─2.96], 0.872
Namayingo		31	26.8	58.8	14.3	0.88 [0.30─2.60], 0.818	0.53 [0.23─1.20], 0.126
Age of the woman^a^							
16–24 years		205	38.2	38.2	23.6	0.61 [0.30–1.21], 0.153	1.26 [0.54–2.96], 0.582
25–29 years		142	31.8	52.7	15.5	Ref	Ref
30–47 years		232	20.5	54.3	25.2	1.60 [0.80–3.21], 0.182	2.52 [0.99–6.41], 0.053
Parity^a^							
1 or 2		192	39.2	36.5	24.3	Ref	Ref
3 or 4		135	18.2	61.8	20.0	3.64 [1.77─7.47], 0.001	1.77 [0.86─3.65], 0.118
5+		252	28.2	49.8	21.9	1.90 [0.95─3.77], 0.068	1.26 [0.73─2.15], 0.400
Residence							
Urban		47	29.7	48.7	21.7	Ref	Ref
Rural		532	29.5	48.1	22.3	0.99 [0.38─2.56], 0.990	1.03 [0.62─1.73], 0.896
Woman’s education level							
No education		47	41.1	41.0	18.0	Ref	Ref
Primary		347	31.3	47.0	21.7	1.50 [0.57─3.94], 0.400	1.59 [0.63─3.99], 0.319
Secondary or higher		185	23.3	52.3	24.4	2.25 [0.78─6.50], 0.132	2.39 [0.81─7.05], 0.111
Woman’s occupation^a^							
Not Employed	161		34.9	35.9	29.3	Ref	Ref
Employed –agriculture	249		27.6	52.5	19.9	1.84 [1.03─3.32], 0.041	0.86 [0.44─1.65], 0.639
Employed –non-agricultural	169		27.3	53.6	19.1	1.91 [0.96─3.82], 0.066	0.83 [0.38─1.83], 0.645
Woman’s religion^a^							
Catholic	94		37.3	46.3	16.5	Ref	Ref
Anglican	205		34.0	43.8	22.1	0.96 [0.47─1.96], 0.919	0.68 [0.21─2.20], 0.512
Muslim	191		26.8	50.7	22.4	1.47 [0.90─2.39], 0.118	1.28 [0.60─2.74], 0.510
Others	89		16.8	54.9	28.3	2.53 [1.09─5.87], 0.031	2.59 [0.95─7.01], 0.062
Marital status							
Never been in union	24		25.7	51.6	22.6	Ref	Ref
Currently in union	516		30.0	46.9	23.0	0.69 [0.31─1.52], 0.349	1.24 [0.43─3.55], 0.689
Formerly in union	40		25.7	62.3	12.0	2.27 [1.13─4.55], 0.202	0.62 [0.23 ─ 1.64], 0.331
Sex of the household head^a^							
Male	451		27.9	50.9	21.2	Ref	Ref
Female	128		35.6	38.5	25.9	0.59 [0.36 ─ 0.97], 0.038	0.96 [0.52 ─ 1.78], 0.885
Wealth index^a^							
Poorest	83		46.7	40.4	12.8	Ref	Ref
Poorer	148		32.0	45.6	22.4	1.65 [1.01 ─ 2.67], 0.044	2.54 [0.98 ─ 6.56], 0.053
Middle	140		26.0	50.3	23.7	2.23 [1.29 ─ 3.88], 0.005	3.32 [1.44 ─ 7.66], 0.006
Richer	138		20.6	55.5	23.9	3.11 [1.69 ─ 5.74], 0.000	4.22 [1.63 ─ 10.88], 0.003
Richest	70		28.7	44.2	27.1	1.78 [0.76 ─ 4.16], 0.179	3.45 [1.58 ─ 7.54], 0.002
Ownership of television or radio							
No	307		25.9	50.7	23.3	Ref	Ref
Yes	253		33.9	45.4	20.7	0.69 [0.41 ─ 1.14], 0.146	0.68 [0.40 ─ 1.15], 0.150
Health seeking decision maker^a^							
Woman alone	153		25.9	47.6	26.5	*Ref*	*Ref*
Woman and partner	177		25.1	56.7	18.2	1.22 [0.59 ─ 2.55], 0.575	0.71 [0.29 ─ 1.72], 0.441
Woman not involved	185		38.1	37.1	24.8	0.53 [0.31 ─ 0.91], 0.022	0.63 [0.30 ─ 1.33], 0.223
Partners age^a^							
< 25 years	88		38.2	38.8	23.1	Ref	Ref
25–35 years	197		37.4	43.1	19.5	1.13 [0.62 ─ 2.06], 0.674	0.86 [0.36 ─ 2.09], 0.737
35 + years	294		21.7	54.4	23.9	2.47 [1.11 ─ 5.52], 0.028	1.83 [0.75 ─ 4.48], 0.184
Partner’s education							
No education	33		38.5	44.6	16.9	Ref	Ref
Primary	289		30.1	48.3	21.6	1.38 [0.59 ─ 3.25], 0.448	1.64 [0.53 ─ 5.03], 0.384
Secondary or higher	184		28.5	45.9	25.6	1.38 [0.45 ─ 4.28], 0.560	2.05 [0.59 ─ 7.14], 0.255
Possession of bed net							
No	113		30.7	53.5	15.9	Ref	Ref
Yes	466		29.3	46.9	23.8	0.92 [0.49 ─ 1.72], 0.790	1.57 [0.84 ─ 2.294], 0.153
Utilization of bed net							
No	194		30.4	53.5	16.0	Ref	Ref
Yes	384		29.1	45.5	25.4	0.89 [0.52 ─1.52], 0.658	1.65 [0.83 ─3.31], 0.152
Fever during pregnancy							
No	162		27.7	48.2	24.1	Ref	Ref
Yes	382		29.8	48.0	22.1	0.92 [0.47 ─1.83], 0.825	0.85 [0.45 ─1.62], 0.622
House sprayed with pesticide in the last 12 months to control malaria							
Not sprayed	537		29.5	47.5	23.0	Ref	Ref
Sprayed	42		30.0	57.4	12.6	1.19 [0.42 ─3.38], 0.741	0.54 [0.11 ─2.51], 0.424
Timing of first ANC visit							
Early ANC^b^	182		23.5	57.6	18.9	Ref	Ref
Late ANC^c^	397		32.3	43.9	23.8	0.55 [0.27 ─1.33], 0.106	0.92 [0.45 ─1.88], 0.807
Number of ANC visits^a^							
Less than 4 times	182		39.5	39.0	21.5	Ref	Ref
4 or more times	397		25.0	52.4	22.6	2.12 [1.25 ─3.60], 0.006	1.67 [0.89 ─3.13], 0.109
ANC provider^a^							
Public/Private hospital	130		15.9	56.1	28.0	Ref	Ref
Lower level health facilities^d^	449		33.5	45.9	20.6	0.39 [0.19 ─ 0.79], 0.010	0.35 [0.15 ─ 0.79], 0.013
Perceived distance to the health facility							
A problem	270		31.8	48.0	20.3	Ref	Ref
Not a problem	309		27.6	48.4	24.0	1.16 [0.65 ─2.06], 0.612	1.36 [0.66 ─2.83], 0.399

^a^Statistically significant (90% level of confidence) at univariate multinomial logistic regression analysis and included in multivariable multinomial logistic regression analysis

^b^Early ANC is the first ANC visit made within the first trimester, that is within 3 months of pregnancy

^c^Late ANC is the first ANC visit made within the second and third trimester, that is from 4th to 9th month of pregnancy

^d^Lower level health facilities that provide ANC services include Health Center IVs Health Center IIIs and private clinics

### Bivariate analysis

Table 3 shows frequency and percentage distribution of uptake of IPTp-SP during pregnancy by the respondents’ characteristics. Age, wealth index, ANC provider, parity, occupation, religion, gender of the household head, woman’s health seeking decision-maker, number of ANC visits, level of health facility where ANC was received and partners age were significantly associated with IPTp-SP uptake.

Receipt of optimal doses of IPTp-SP was significantly more likely to be highest among older women (at least 30 years) compared to women age 25–29 years (25.2% *versus* 15.5%) and among women from households in the middle (23.7% *versus* 12.8%), richer (23.7% *versus* 12.8%) and richest (27.9% *versus* 12.8%) wealth indices compared to the poorest wealth index.

Women with parity of 3 or 4 were significantly more likely to receive partial dose of IPTp-SP compared to women with a parity of 1 or 2 (61.8% *versus* 36.5%) as well as women employed in agriculture compared to unemployed women (52.5% *versus* 35.9%). Partial uptake of IPTp-SP was also significantly higher among women with older (at least 35 years) partners relative to women with younger (under 25 years) partners (54.4% *versus* 38.8%) and among women from male headed households relative to women from female headed households (50.9% *versus* 38.5%). Additionally, partial uptake of IPTp-SP was significantly higher among women from households in poorer (45.6% *versus* 40.4%), middle (50.3% *versus* 40.4%) and richer (55.5% *versus* 40.4%) wealth indices compared to the poorest wealth index. Furthermore, partial uptake of IPTp-SP was significantly higher among women who were involved in decision making about seeking their healthcare compared to women who were not (47.6% *versus* 37.1%). Lastly, there was no significant differences observed in partial or optimal uptake of IPTp-SP between Anglican and Muslim compared to Catholic women. However, partial uptake of IPTp-SP was statistically significantly lower among Catholic women relative to women from other religions denominations (54.9% *versus* 46.3%). Other religious denominations included 7th day Adventists (10%), a combination of Pentecostals, Born-agains and Evangelicals (85.9%) and the undefined others (3.9%).

### Antenatal care

**Fig. 2:**
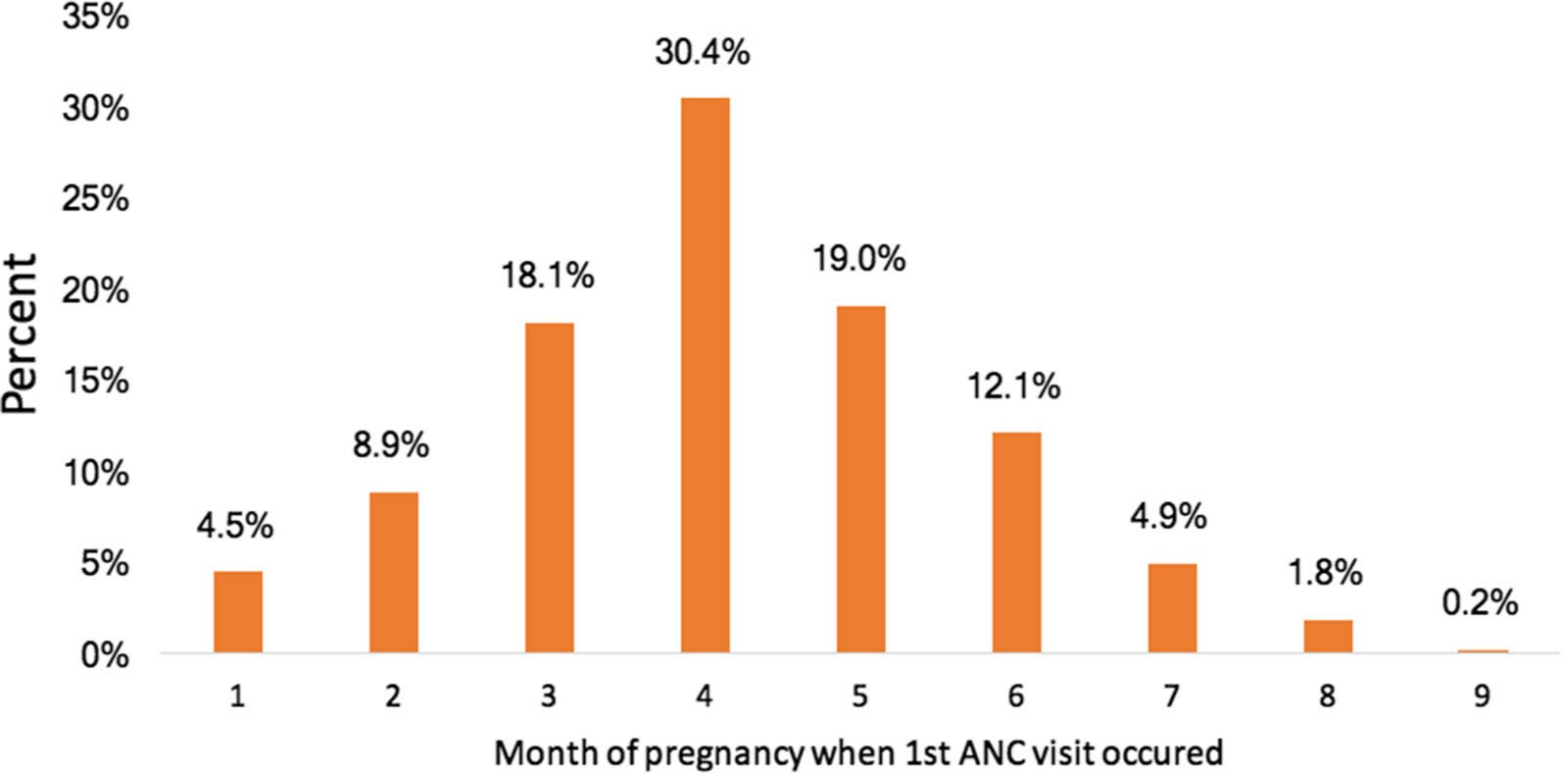
Percentage of women by month of first ANC visit

**Fig. 3:**
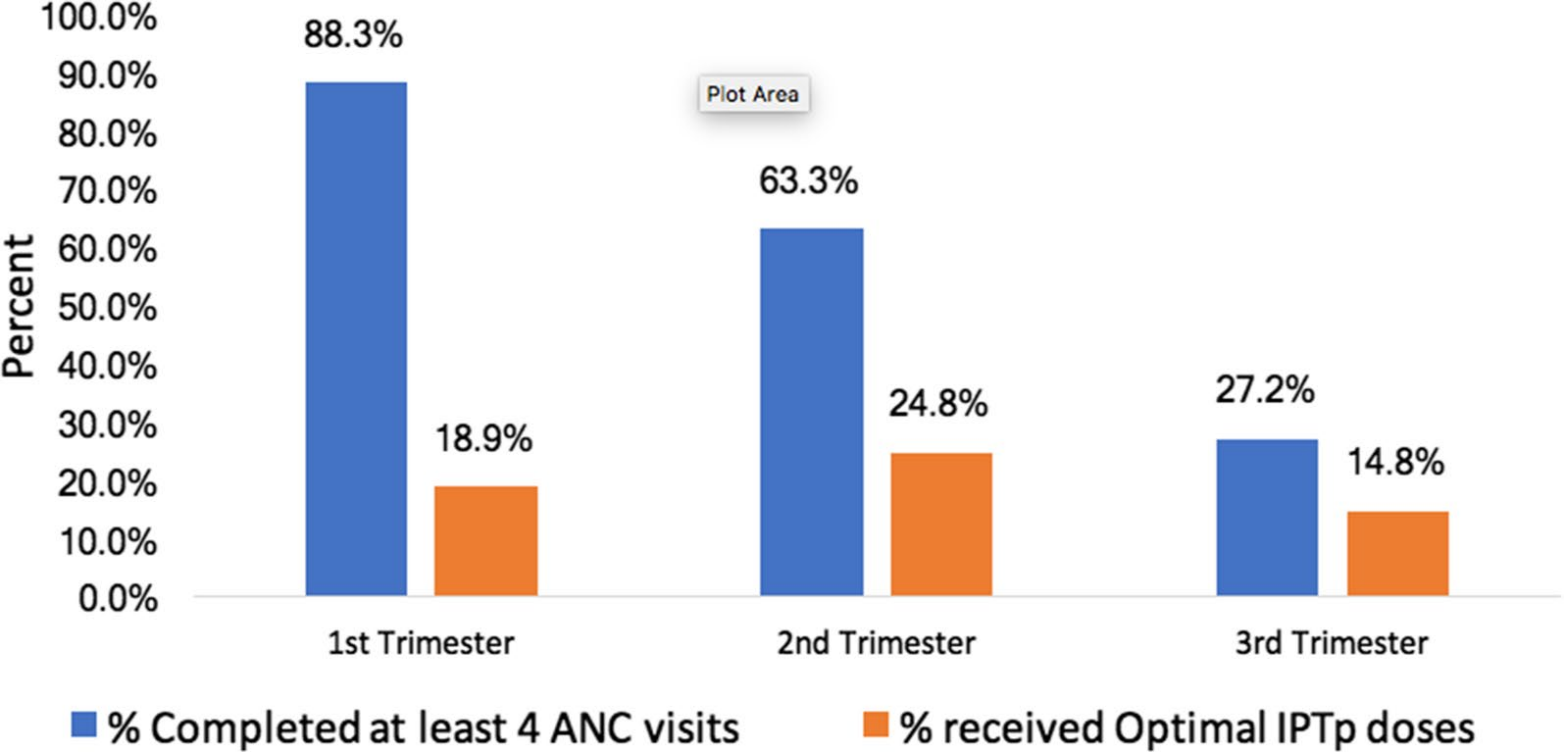
Difference between completing at least 4 ANC visits and receiving optimal doses of IPTp-SP

Uptake of partial dose of IPTp-SP was higher among pregnant women who attended ANC at least four times (52.4% *versus* 39.0%, p = 0.006). Figure 2 shows that majority (30.4%) of the women made their first ANC visit in the 4th month. Figure 3 shows that, among women who made their first ANC visit in the first trimester, 88.3% went ahead and completed at least four ANC visits, but only 18.9% received optimal doses of IPTp-SP.

The ANC provider was significantly associated with uptake of partial (p = 0.010) and optimal (p = 0.013) doses of IPTp-SP. Uptake of partial (56.1% *versus* 45.6%) and optimal (28.0% *versus* 20.6%) dose of IPTp-SP was higher among pregnant women who received ANC from a hospital than a lower level health facility.

### Multivariate analysis

The results from the multivariate multinomial logistic regression analysis of factors associated with optimal and partial uptake of IPTp-SP are presented in Table 4. The factors significantly associated with both optimal and partial uptake of IPTp-SP in East-Central Uganda were place of ANC and religious faith. Women who received ANC from lower level health facilities had a 69% less likelihood of receiving optimal doses of IPTp-SP (RRR = 0.31, 95%CI 0.10–0.94, *p* value = 0.039) and 66% less likelihood of receiving partial doses of IPTp-SP (RRR = 0.33, 95%CI 0.15–0.77, p-value = 0.011) than women who received ANC from hospitals. Belonging to other religious denominations was significantly associated with a 3.68 and 3.42 times increased likelihood of receiving optimal (RRR = 3.68, 95%CI 1.28–10.6, p-value = 0.016) and partial (RRR = 3.42, 95%CI 1.45–8.04, p-value = 0.006) doses of IPTp-SP respectively during pregnancy. The other religious denominations mainly included Evangelicals, Born-agains, Pentecostals and 7th-day Adventists.

**Table 4:** Multivariate multinomial logistic regression models of correlates of IPTp-SP uptake in East Central Uganda, (n = 579)

Variable	IPTp-SP Uptake			
	Partial versus none		Optimal versus none	
	Relative risk ratio (RRR) [95% CI]	P-value	Relative risk ratio (RRR) [95% CI]	P-value
Woman’s age				
25–29 years (Ref)				
16–24 years	0.73 [0.24–2.27]	0.583	1.30 [0.35–4.83]	0.690
30–47 years	1.94 [0.79–4.78]	0.144	3.94 [1.16–13.34]	0.028**
Parity				
1 or 2 (Ref)				
3 or 4	3.45 [1.62–7.32]	0.002**	1.81 [0.87–3.75]	0.112
5+	0.95 [0.36–2.50]	0.910	0.56 [0.20–1.58]	0.271
Woman’s occupation				
Unemployed (Ref)				
Employed in agriculture	1.45 [0.73–2.86]	0.280	0.77 [0.32–1.82]	0.537
Employed in non-agriculture	1.10 [0.48–2.52]	0.810	0.41 [0.15–1.13]	0.083*
Religion				
Catholic (Ref)				
Anglican	1.05 [0.55–1.98]	0.891	0.69 [0.24–1.96]	0.477
Muslim	1.68 [1.01–2.78]	0.045**	1.46 [0.71–2.99]	0.297
Others	3.19 [1.37–7.41]	0.008***	3.35 [1.25–8.97]	0.017**
Sex of the household head				
Male (Ref)				
Female	0.55 [0.29–1.05]	0.070*	0.85 [0.43–1.67]	0.624
Wealth index				
Poorest (Ref)				
Poorer	1.25 [0.60–2.57]	0.546	2.04 [0.65–6.44]	0.220
Middle	1.56 [0.78–3.11]	0.203	3.07 [1.05–8.98]	0.041**
Richer	1.97 [0.79–4.88]	0.141	3.12 [0.95–10.26]	0.061*
Richest	0.80 [0.27–2.35]	0.677	1.98 [0.46–8.59]	0.357
Health seeking decision maker				
Woman alone (Ref)				
Woman and partner	1.03 [0.34–1.68]	0.934	0.59 [0.21–1.66]	0.313
Woman not involved	0.52 [0.61–0.96]	0.038**	0.58 [0.25–1.33]	0.193
Partner’s age				
<25 years (Ref)				
25–34 years	0.76 [0.34–1.68]	0.487	0.79 [0.28–2.26]	0.656
<35 years	1.54 [0.48–2.52]	0.350	1.43 [0.57–3.58]	0.433
ANC provider				
Hospital (Ref)				
Lower level health facility^a^	0.33 [0.15–0.76]	0.010***	0.29 [0.10–0.91]	0.033**
Number of ANC visits				
<4 ANC visit (Ref)				
At least 4 ANC visits	2.10 [1.14–3.86]	0.019**	1.65 [0.97–2.83]	0.065*

R*ef* reference category

***P < 0.001, **P < 0.050, *P < 0.100

^a^Lower level health facilities that provide ANC services include Health Center IVs Health Center IIIs and private clinics

Two covariates were significantly associated with uptake of optimal but not partial doses of IPTp-SP during pregnancy include: wealth index and place of child delivery. Women from households in the middle wealth index were 3.10 more likely to receive optimal doses of IPTp-SP that women from households in the poorest wealth index (RRR = 3.10, 95%CI 1.09, 8.76, p-value = 0.034). Women that delivered from outside of a health facility had a 61% less likelihood of receiving an optimal dose of IPTp-SP compared to women that eventually delivered from within a health facility (RRR = 0.39, 95%CI 0.17–0.87, p-value = 0.022).

Four covariates were also found to be significantly associated with uptake of partial but not optimal doses of IPTp-SP during pregnancy. These were: being a Muslim, parity, health seeking decision making and number of ANC visits. Muslim women were 1.73 times more likely to receive partial dose of IPTp-SP than Catholic women (RRR = 1.73, 95% CI 1.03–2.90, p-value = 0.038). Women of parity of 3 or 4 pregnancies were 3.09 times more likely to receive a partial dose of IPTp-SP than women of parity 1 or 2 pregnancies (RRR = 3.09, 95% CI 1.42–6.75, p-value = 0.005). Women from households in which they are not involved in making decisions on seeking health had 48% less likelihood of receiving a partial dose of IPTp-SP than women from households in which they were the sole decision makers (RRR = 0.52, 95% CI 0.28–0.95, p-value = 0.034). Women who attended at least four ANC visits were 2.05 times more likely to receive a partial dose of IPTp-SP (RRR = 2.05, 95%CI 1.12–3.74, p-value = 0.021) than women who attended ANC less than four times.

## Discussion

In this study, the coverage of at least three doses of IPTp-SP in East-Central Uganda was 22.3%, which is far below the 93% that Uganda targets to achieve by 2019/20 [40]. This coverage however has potential to increase to at least 70% if the 48.2% of women that received a partial dose sustained uptake of IPTp-SP.

The low uptake of optimal doses of IPTp-SP is indicative of a weak health system and sub-optimal implementation of the IPTp-SP policy which remains a challenge in eradication of malaria in pregnancy, in Uganda and other low-income countries. The low uptake could be attributed to several barriers including widespread stock-outs of SP [41–43], limited knowledge of the IPTp-SP guidelines among health workers [44], lack of awareness of the benefits of IPTp-SP amongst pregnant women [45] and complacency of health care providers in ensuring that women who attend ANC complete the optimal IPTp-SP dosage [44]. To improve IPTp-SP coverage, there is a pertinent need for capacity building of health workers to address the supply side gaps. Additionally, the demand side gaps can be addressed through social behaviour change interventions including health education sessions at health facilities, media campaigns and community engagement during outreach activities at the community level in order to shape women’s and community attitudes towards IPTp [26, 27].

The findings showed that the factors associated with optimal uptake IPTp-SP in East-Central Uganda were religion, age, wealth status, level of health facility where ANC was received and place of delivery. It was revealed that women from other religious groups were more likely to receive optimal doses of IPTp-SP than Catholics. This is similar to findings in Malawi which showed that Catholic/Anglican women were less likely to complete at least 3 doses of IPTp-SP during pregnancy relative to women of other religions denominations, that is Baptists, Church of Central Africa Presbyterian and Seventh Day Adventists [33]. There is paucity of information that explains the low uptake of IPTp-SP among Catholic women especially in Uganda, which calls for further research. This finding calls for more involvement of Catholic religious leaders in promoting uptake of IPTp-SP among women followers. Some studies have demonstrated improved uptake of health services when religious leaders were involved in health promotion [46, 47].

The likelihood of completing optimal doses of IPTp-SP was statistically significantly higher among older women 30 years and above than younger women aged 25–30 years. This result compares to other similar results in Malawi [33, 48]. It is therefore imperative that interventions to increase uptake of optimal doses of IPTp-SP among pregnant women focus on 25–30 years population group.

Further, women from households in the middle wealth index had a significantly increased likelihood of receiving optimal doses of IPTp-SP than women from poorest households. Although only the middle wealth index was statistically significant, results showed that generally, completion of at least 3 doses of IPTp-SP increased with wealth. This finding agrees with findings of a study conducted among pregnant women from eight sub-Saharan African countries where IPTp-SP uptake was significantly associated with household wealth quintile [16]. It is possible that poor women are less educated and may not easily access information and even when they do, they may not easily appreciate it [49]. Poverty has also been found to be associated with poor health-seeking behaviours [50].

Level of health facility where ANC was received was significantly related to uptake of IPTp-SP with those receiving ANC at lower level health facilities reporting lower uptake than those who received ANC at hospitals. This finding is in consonance with findings elsewhere which indicated that quality of basic maternal health services was poorer or absent in lower level facilities [26, 27, 51]. Poor uptake at this level of health facilities could possibly be due to non-adherence to protocols, stock-outs of SP, overwhelming patient load and understaffing as reported [26, 27].

Although the effect of attending at least four ANC visits was not significantly associated with uptake of IPTp-SP, the relative risk ratio was positive indicating a benefit of higher ANC visits on increasing women’s uptake of the optimal doses. The results however, show that ANC was significantly associated with partial uptake of IPTp-SP. Findings in this study indicate that although completion of at least four ANC visits was higher among women who made their first ANC visit in the first trimester (nearly 90%) compared to second trimester (63%), receiving optimal doses of IPTp-SP was surprisingly lower among women whose first ANC visit was in the first trimester (18.9%) compared to the second trimester (24.8%). This highlights the many missed opportunities when IPTp-SP could have been provided especially among women who come early for ANC and calls for further research to understand the reasons for this anomaly.

Although not a major focus of this study, factors associated with uptake of partial doses of IPTp-SP were also established. Parity of 3 or 4 children relative to fewer (1 or 2) children, being Muslim relative to Catholic, and attending at least four ANC visits were associated with higher likelihood of receiving partial doses of IPTp-SP. On the contrary, non-involvement of a woman in decision making about seeking their healthcare was associated with reduced likelihood of receiving partial doses of IPTp-SP. Literature has shown that women with higher parity tend to utilize maternal health services more frequently compared to women with fewer children [52]. Strengthening interventions around factors associated with increased uptake of partial doses could in turn improve uptake of optimal doses of IPT-SP. Therefore, ensuring mothers of low parity appreciate the importance of completing optimal doses of IPTp-SP, involving women in decision-making about seeking their own healthcare [53] and ensuring that more women complete at least four ANC visits may increase uptake of IPTp-SP.

### Study limitations

These results were liable to recall bias and social desirability bias since it was a self-reported survey and use of IPTp-SP in pregnancy is considered desirable behaviour. However, such bias was minimized to some extent by restricting the study to mothers with a birth within 2 years of the survey. In addition, all study results relate to the most recent birth. Additionally, data on factors that would have been useful in explaining the poor uptake of optimal doses of IPTp-SP such as timing of the first dose of IPTp-SP, availability of SP and items for facilitating Directly Observed Treatment, such as cups and clean drinking water at the health facilities, and women’s perception and knowledge related to IPTp-SP were not collected during the survey. Despite these limitations, this report provides useful information for the assessment and implementation of IPTp-SP policy and will also inform policy decisions in Uganda and other countries of comparable economic status.

### Conclusions and recommendations

This study has demonstrated low coverage of the WHO-recommended optimal doses of IPTp-SP for prevention of malaria during pregnancy. There is need to strengthen ANC services especially at lower level health centres, including early ANC attendance, of at least four ANC visits and leveraging increased ANC attendance to increase demand for IPTp-SP services. Factors associated with uptake of IPTp-SP varied across various sectors. Therefore, sector-wide approaches, including female education, poverty eradication, involving women in decision-making about seeking their own healthcare and engaging religious institutions in malaria control efforts are needed to scale-up IPTp-SP uptake. This study highlights significant low IPTp-SP uptake especially among women aged under 30 years and women who come early for ANC, but yet complete at least four ANC visits and women who attend ANC at lower level health facilities. Therefore, there is need for implementation research to understand implementation challenges that explain the poor prescription practices of IPTp-SP at lower level health facilities and among younger women.

## Data Availability

The study used obtained with permission from ICF International. The full 2016 UDHS dataset is publicly available and may be requested from ICF on (https://dhsprogram.com/data/dataset/Uganda_Standard-DHS_2016.cfm?flag=0). The woman questionnaire used to collect the data that were analyzed in this manuscript can be accessed publicly at (http://microdata.worldbank.org/index.php/catalog/2979/download/41502).
